# The role of open abdomen in non-trauma patient: WSES Consensus Paper

**DOI:** 10.1186/s13017-017-0146-1

**Published:** 2017-08-14

**Authors:** Federico Coccolini, Giulia Montori, Marco Ceresoli, Fausto Catena, Ernest E. Moore, Rao Ivatury, Walter Biffl, Andrew Peitzman, Raul Coimbra, Sandro Rizoli, Yoram Kluger, Fikri M. Abu-Zidan, Massimo Sartelli, Marc De Moya, George Velmahos, Gustavo Pereira Fraga, Bruno M. Pereira, Ari Leppaniemi, Marja A. Boermeester, Andrew W. Kirkpatrick, Ron Maier, Miklosh Bala, Boris Sakakushev, Vladimir Khokha, Manu Malbrain, Vanni Agnoletti, Ignacio Martin-Loeches, Michael Sugrue, Salomone Di Saverio, Ewen Griffiths, Kjetil Soreide, John E. Mazuski, Addison K. May, Philippe Montravers, Rita Maria Melotti, Michele Pisano, Francesco Salvetti, Gianmariano Marchesi, Tino M. Valetti, Thomas Scalea, Osvaldo Chiara, Jeffry L. Kashuk, Luca Ansaloni

**Affiliations:** 1 General, Emergency and Trauma Surgery dept., Papa Giovanni XXIII Hospital, Piazza OMS 1, 24127 Bergamo, Italy; 2 Emergency and Trauma Surgery, Parma Maggiore hospital, Parma, Italy; 3 Denver Health, Denver, CO 80204 USA; 4 Trauma Surgery, Virginia Commonwealth University, Richmond, VA 23284 USA; 5 Acute Care Surgery, The Queen’s Medical Center, Honolulu, HI 96813 USA; 6 Department of Surgery, Trauma and Surgical Services, University of Pittsburgh School of Medicine, Pittsburgh, 15213 USA; 7 Department of Surgery, UC San Diego Health System, San Diego, 92103 USA; 8 Trauma & Acute Care Service, St Michael’s Hospital, Toronto, ON Canada; 9 Division of General Surgery Rambam Health Care Campus, Haifa, Israel; 10 Department of Surgery, College of Medicine and Health Sciences, UAE University, Al-Ain, United Arab Emirates; 11 Department of Surgery, Macerata Hospital, Macerata, Italy; 12 Department of Trauma, Emergency Surgery and Surgical Critical Care, Massachusetts General Hospital, Boston, MA 02114 USA; 13 Faculdade de Ciências Médicas (FCM) – Unicamp Campinas, São Paulo, Brazil; 14 Second Department of Surgery, Meilahti Hospital, Helsinki, Finland; 15 Academic Medical Center Amsterdam, Amsterdam, The Netherlands; 16 Department of Surgery, Foothills Medical Centre, Calgary, Canada; 17 Department of Surgery, Harborview Medical Centre, Seattle, 98104 USA; 18 General Surgery Department, Hadassah Medical Centre, Jerusalem, Israel; 19 First Clinic of General Surgery, University Hospital/UMBAL/St George Plovdiv, Plovdiv, Bulgaria; 20 General Surgery, Mozir Hospital, Mozir City, Belarus; 21 ICU and High Care Burn Unit, Ziekenhius Netwerk Antwerpen, Antwerpen, Belgium; 22 ICU Department, Bufalini Hospital, Cesena, Italy; 23 Critical Care Centre, Corporasiò Sanitaria Park Tauli, Sabdel, Spain; 24 General Surgery Department, Letterkenny Hospital, Letterkenny, Ireland; 25 General and Trauma Surgery Department, Maggiore Hospital, Bologna, Italy; 26 Upper Gatrointestinal Surgery, Birmigham Hospital, Birmigham, UK; 27 Department of Gastrointestinal Surgery, Stavanger University Hospital, Stavanger, Norway; 28 Department of Clinical Medicine, University of Bergen, Bergen, Norway; 29 Department of Surgery, School of Medicine, Washington University, Saint Louis, MO 63130 USA; 30 Departments of Surgery and Anesthesiology, Division of Trauma and Surgical Critical Care, Vanderbilt University Medical Center, Nashville, TN 37232 USA; 31 Département d’Anesthésie-Réanimation, CHU Bichat Claude-Bernard-HUPNVS, Assistance Publique-Hôpitaux de Paris, University Denis Diderot, Paris, France; 32 ICU department Sant’Orsola-Malpighi University Hospital, Bologna, Italy; 33 ICU Department, Papa Giovanni XXIII Hospital, Bergamo, Italy; 34 Trauma Surgery department, University of Maryland School of Medicine, Baltimore, MD 21201 USA; 35 Emergency and Trauma Surgery department, Niguarda Hospital, Milan, Italy; 36 General Surgery department, Assuta Medical Centers, Tel Aviv, Israel

**Keywords:** Open abdomen, Laparostomy, Non-trauma, Peritonitis, Pancreatitis, Vascular emergencies, Fistula, Nutrition, Re-exploration, Re-intervention, Closure, Biological, Synthetic, Mesh, Technique, Timing

## Abstract

The open abdomen (OA) is defined as intentional decision to leave the fascial edges of the abdomen un-approximated after laparotomy (laparostomy). The abdominal contents are potentially exposed and therefore must be protected with a temporary coverage, which is referred to as temporal abdominal closure (TAC). OA use remains widely debated with many specific details deserving detailed assessment and clarification. To date, in patients with intra-abdominal emergencies, the OA has not been formally endorsed for routine utilization; although, utilization is seemingly increasing. Therefore, the World Society of Emergency Surgery (WSES), Abdominal Compartment Society (WSACS) and the Donegal Research Academy united a worldwide group of experts in an international consensus conference to review and thereafter propose the basis for evidence-directed utilization of OA management in non-trauma emergency surgery and critically ill patients. In addition to utilization recommendations, questions with insufficient evidence urgently requiring future study were identified.

## Background

The decision by a surgeon to utilize the open abdomen (OA) technique is a dramatically non-anatomic situation that dramatically increases resource utilization and has potential severe side effects. It is, however, often dramatically effective at countering the drastically impaired physiology of critical illness when no other perceived options exist. There are both mandatory and relative indications for OA use, which are heavily influenced by the primary pathophysiologic insults and responses to intra-abdominal sepsis and inflammation, both inherent to the patient and induced through medical treatments. The abdominal compartment is dramatically affected in both its contents and the characteristics of the abdominal wall. Several factors as systemic inflammatory response syndrome, increased vascular permeability, and aggressive crystalloid resuscitation predispose to fluid sequestration leading to peritoneal fluid formation. Patients with severe sepsis and septic shock commonly receive large amounts of resuscitation fluids and may develop excessive gut edema and diminished contractility and motility. These changes in combination with sequestration of second and third space fluids and forced closure of an abdominal wall with altered compliance may result in increased intra-abdominal pressure (IAP) ultimately leading to intra-abdominal hypertension (IAH) or even abdominal compartment syndrome (ACS) [1, 2].

The pathophysiologic implications of elevated IAP have been restarted to be studied in deep during the last 20 years [2–4]. In 2013, The Abdominal Compartment Society (WSACS) updated the previously published definition and guidelines for the management of intra-abdominal hypertension [5]. Elevated IAP constitutes IAH and was classified into four grades: (1) grade I IAP 12–15 mmHg, (2) grade II IAP 16–20 mmHg, (3) grade III IAP 21–25 mmHg, and (4) grade IV IAP >25 mmHg. Elevated IAP commonly causes marked deficits in loco-regional and whole body perfusion that may result in organ failure [5]. An uncontrolled IAH, with an IAP exceeding 20 mmHg and new onset organ failure, is defined as an abdominal compartment syndrome (ACS) [2, 5]. ACS is a syndrome and not a disease, as such, it can have many causes and it can occur in many disease processes, it is an all or nothing phenomenon, while IAH is a more graded continuum. ACS in turn has further effects on intra-abdominal organs, as well as indirect effects on the other organ(s) and system(s). The ACS is a potentially and frequently lethal complication characterized by effects on splanchnic, cardiovascular, pulmonary, renal, and central nervous systems [2, 5]. While medical therapies should be attempted, the ACS is rapidly lethal and opening of the abdominal cavity conducted promptly if medical interventions do not quickly alleviate or temporize the situation. If surgery has been undertaken for the index disease, leaving the abdomen temporarily open is often required to prevent inducing ACS in a critically ill pro-inflammatory patient with visceral edema and ongoing resuscitation. Whether leaving the abdomen open will primarily influence the septic response is also intriguing but unproven at the present time.

The OA procedure is defined as intentionally leaving the fascial edges of the abdomen un-approximated (laparostomy). The abdominal contents are exposed and thus must be protected with a temporary coverage, which is itself termed a temporary abdominal coverage (TAC) [2, 6]. The OA technique, when used appropriately, may be useful in the management of surgical patients with compromised general conditions due to any critical illness/injury but most frequently cases of intra-abdominal sepsis and severe pancreatitis are seen recently [7]. Despite many serious potential complications, the OA is perceived to be a life-saving intervention in catastrophically injured patients [2]. Compared to trauma patients, however, patients undergoing OA management for intra-abdominal non-trauma emergencies have greater risks subsequent to OA utilization, including entero-atmospheric fistula (EAF) and a “frozen abdomen”, intra-abdominal abscesses, and lower rates of definitive fascial closure [8, 9] with resultant large ventral hernia defects. This discrepancy in risks and benefits, along with economic considerations [10], was the primary reason the WSACS suggested not routinely using the OA for septic cases versus traumatic cases [5]. Thus, every effort should be exerted to attempt abdominal closure as soon as the patient can physiologically tolerate it.

## Methods

The recommendations are formulated and graded according to the *modified Grading of Recommendations Assessment*, *Development and Evaluation* (GRADE) hierarchy of evidence from the GRADE Group, summarized in the Table 1 [11].

**Table 1:** “Modified Grading of Recommendations Assessment, Development and Evaluation (GRADE)” hierarchy of evidence from the American College of Chest Physicians task force by Guyatt and colleagues [11]

Grade of recommendation	Clarity of risk/benefit	Quality of supporting evidence	Implications
1A			
Strong recommendation, highquality evidence	Benefits clearly outweigh risk and burdens, or vice versa	RCTs without important limitations or overwhelming evidence from observational studies	Strong recommendation, applies to most patients in most circumstances without reservation
1B			
Strong recommendation, moderate-quality evidence	Benefits clearly outweigh risk and burdens, or vice versa	RCTs with important limitations (inconsistent results, methodological flaws, indirect analyses or imprecise conclusions) or exceptionally strong evidence from observational studies	Strong recommendation, applies to most patients in most circumstances without reservation
1C			
Strong recommendation, lowquality or very lowquality evidence	Benefits clearly outweigh risk and burdens, or vice versa	Observational studies or case series	Strong recommendation but subject to change when higher quality evidence becomes available
2A			
Weak recommendation, high-quality evidence	Benefits closely balanced with risks and burden	RCTs without important limitations or overwhelming evidence from observational studies	Weak recommendation, best action may differ depending on the patient, treatment circumstances, or social values
2B			
Weak recommendation, moderate-quality evidence	Benefits closely balanced with risks and burden	RCTs with important limitations (inconsistent results, methodological flaws, indirect or imprecise) or exceptionally strong evidence from observational studies	Weak recommendation, best action may differ depending on the patient, treatment circumstances, or social values
2C			
Weak recommendation, Low-quality or very lowquality evidence	Uncertainty in the estimates of benefits, risks, and burden; benefits, risk, and burden may be closely balanced	Observational studies or case series	Very weak recommendation; alternative treatments may be equally reasonable and merit consideration

The WSES and Abdominal Compartment Society together with the Donegal Research Academy united a group of subject-matter experts coordinated by a central coordinator to review and summarize the evidence and thereafter to express their evidence-based opinion on important issues concerning OA utilization in non-trauma patients:

Which non-trauma patients can benefit from OA techniques and for which specific critical conditions is indicated (example, peritonitis, vascular emergencies, and severe pancreatitis)?

What is the optimum TAC technique for use in non-trauma patients?

Is there a role for fluid instillation?

What is the optimum timing of re-exploration before definitive closure in non-trauma patients?

What is the optimum timing to definitively close an OA in non-trauma patients?

What are the optimum adjunctive techniques to definitively close an OA in non-trauma patients considering both non-mesh-mediated techniques and mesh-mediated techniques?

What is the optimum treatment to treat frozen abdomen and enteral fistulas?

What nutritional support is indicated in OA?

A central project coordinator compiled the answers and statements derived from the first round of presentations and discussions. The statements were discussed during the Consensus Conference held in Dublin (Ireland) in July 2016. Once an agreement was reached within the experts groups, a final round of discussion among a larger group of experts led to the final version of recommendations reflecting the final expert-consensus document (Table 2).

**Table 2:** Statement Grid

	Statements
Open Abdomen indication:	
➢ Peritonitis	The open abdomen is an option for emergency surgery patients with severe peritonitis and septic shock under the following circumstances: abbreviated laparotomy due to the severe physiological derangement, or the need for a deferred intestinal anastomosis or a planned second look for intestinal ischemia, or persistent source of peritonitis (failure of source control), or extensive visceral edema with the concern for development of abdominal compartment syndrome (Grade 2C).
➢ Vascular Emergencies	The open abdomen should be strongly considered following management of hemorrhagic vascular catastrophes such as ruptured abdominal aortic aneurysm (Grade 1C) The open abdomen should be considered following surgical management of acute mesenteric ischemic insults (Grade 2C).
➢ Pancreatitis	In patients with severe acute pancreatitis unresponsive to step-up conservative management surgical decompression and leaving the abdomen open is effective in treating abdominal compartment syndrome (Grade 2C) Leaving the abdomen open after surgical necrosectomy for infected pancreatic necrosis is not recommended excepted in those situation at high risk of abdominal compartment syndrome (Grade 1C)
Optimal technique for temporary abdominal closure	Negative pressure wound therapy with continuous fascial traction is suggested as the preferred technique for temporary abdominal closure (Grade 1B). Temporary Abdominal Closure without Negative pressure wound therapy (e.g., mesh alone, Bogota bag) whenever possible should NOT be applied for the purpose of temporary abdominal closure, because of low delayed fascial closure rate and being accompanied by a significant intestinal fistula rate (Grade 1B).
*Is there a role for NPWT with Fluid Instillation?*	There is inadequate evidence to make a recommendation regarding use of negative pressure wound therapy in combination with fluid instillation in patients with temporary abdominal closure (NOT GRADED).
Planning re-exploration before definitive closure	- In critically ill non-trauma patients with open abdomen, once any requirements for on-going resuscitation have ameliorated, early re-operation with the intention of closing the abdomen should be given a high priority (Grade 1C). - In critically ill patients with open abdomen, re-laparotomy with concern for ongoing ischemia/contamination reoperation should be conducted no later than 24–48 h after the index operation, with the duration from the index operation shortening with increasing degrees of patient non-improvement and hemodynamic instability (Grade 1C).
Best timing to definitively close an open abdomen	- Fascia should be closed as soon as possible (Grade 1C). - Acidosis (pH <7.25), hypothermia (temperature < 34 °C) and coagulopathy (TEG, INR) are not predictive of the need for maintaining the open abdomen in non-trauma patients (Grade 2A). - The abdomen should be maintained open in non-trauma patients if the source of contamination persists, if a condition of haemodynamic instability persists meaning in presence of on-going fluid resuscitation or vasopressor support necessity, if a deferred intestinal anastomosis is needed, if there is the necessity for a planned second look for ischemic intestine and lastly if there are concerns about abdominal compartment syndrome development (Grade 2C). - Early fascia closure (within 7 days) should be the strategy for management of the open abdomen once the source control has been reached, the severe sepsis has been controlled meaning that the patient is haemodynamically stable and the hypoperfusion has been definitively corrected, no further surgical re-exploration is needed and there are no concerns for abdominal compartment syndrome (Grade 2C).
Best solution to definitively close an open abdomen	
➢ *Non-mesh mediated techniques*	- Primary fascia closure is the ideal solution to restore the abdominal closure (2A). - Component separation is an effective technique; however, it’s early use is NOT recommended in fascial temporary closure. It should be considered only for definitive closure or reconstructive interventions (Grade 2C) - Planned ventral hernia (skin graft or skin closure only) remains an option for complicated open abdomen (i.e. in the presence of entero-atmospheric fistula or in cases with a protracted open abdomen due to underlying diseases) or in those low resource setting where no other facilities are present (Grade 2C)
➢ *Mesh mediated techniques*	- A fascial bridge using prosthetic mesh (polypropylene, polytetrafluoruroethylene (PTFE) and polyester products) should NOTt be recommended to achieve definitive fascial closure in patients with open abdomen and should be placed only in patients without other alternatives (Grade 1B). - Biologic meshes are reliable for definitive abdominal wall reconstruction in the presence of a large wall defect, bacterial contamination, comorbidities and difficult wound healing. NPWT can be used combined with biologic mesh to facilitate granulation and skin closure (Grade 2B). - Non–cross-linked biologic meshes seem to be preferred in sublay position when the linea alba can be reconstructed. Non–cross-linked biologic mesh is easily integrated, with reduced fibrotic reaction and lesser infection and removal rate (Grade 2B). - The long-term outcome of a bridging non–cross-linked biologic mesh is laxity of the abdominal wall and a high rate of recurrent ventral hernia. In the bridge position (no linea alba closure), cross-linked biologic meshes maybe associated with less ventral hernia recurrence (Grade 2B).
Best treatment for open abdomen and entero-atmospheric fistulas	- Several clinical circumstances may contribute to the development of entero-atmospheric fistula and few risk factors may predict its development. Awareness of this complication and avoidance of contributing conditions for its development are mandatory; moreover preemptive measures are imperative (Grade 1C). - The management of entero-atmospheric fistula should be personalized according to standard classification and grading system. Current different classification schemes echo the problematic and challenging issues related to their management (Grade 1C) - The caloric intake and protein demands of patients with entero-atmospheric fistula increase; the Nitrogen balance should be corrected and protein supplemented. Nutrition should be started immediately upon recognition of entero-atmospheric fistula (Grade 1C) - Entero-atmospheric fistula effluent isolation is essential for proper wound healing. Separating the wound into different compartments in order to facilitate the collection of fistula output is of paramount importance (Grade 2A). - Many methods for wound care exist; however in the presence of entero-atmospheric fistula in open abdomen, negative pressure wound therapy makes effluent isolation feasible and wound healing conceivable (Grade 2A). Definitive management of entero-atmospheric fistula should be delayed to after the patient has recovered and the wound completely healed (Grade 1C).
Nutritional support	- Open abdomen patients are in a hyper-metabolic condition; an immediate and adequate nutritional support is mandatory (Grade 1C). - Open abdomen techniques result in a significant nitrogen loss that must be replaced with a balanced nutrition regimen (Grade 1C). - Early enteral nutrition should be started as soon as possible if the gastrointestinal tract allows (Grade 1C). - Enteral nutrition should be delayed in patients with high output fistula with no possibility to obtain feeding access distal to the fistula (Grade 2C) - Oral feeding is not contraindicated; whenever it’s possible it could be started as soon as the patient is able to eat (Grade 2C).
Patient Mobilization	- To date, no recommendations can be made about early mobilization of patients with open abdomen.

### Open abdomen in peritonitis

*The open abdomen is an option for emergency surgery patients with severe peritonitis and septic shock under the following circumstances*: *abbreviated laparotomy due to the severe physiological derangement*, *or the need for a deferred intestinal anastomosis or a planned second look for intestinal ischemia*, *or persistent source of peritonitis (failure of source control)*, *or extensive visceral edema with the concern for development of abdominal compartment syndrome* (*grade 2C*).

In severe secondary peritonitis, some patients may experience a disease progression to severe sepsis and septic shock experiencing progressive organ dysfunction, hypotension, myocardial depression, and coagulopathy and a staged approach may be required [12]. These are often hemodynamically unstable and unfit for immediate complex surgical interventions [12]. If the patient is not in a condition to be undergone to a definitive repair and/or abdominal wall closure, the intervention should be abbreviated due to suboptimal local conditions for healing and global susceptibility to spiraling organ failure. For instance, intestinal continuity restoration can be deferred to a subsequent surgical intervention, which is particularly important in hypotensive patients who are receiving inotropes [13]. In facing the impossibility to completely obtain a source control of the contamination in a single operation or if extensive visceral edema and decreased abdominal wall compliance increases the risk of ACS development, primary fascial closure should not be attempted and the abdomen should be left open [14]. The rationale for using the OA is to leave the abdomen open and to treat the infected peritoneal cavity like an “open abscess” with subsequent re-operations involving generous irrigations and potentially active TAC techniques [15] to definitively control the contamination while also preventing IAH progression to ACS. No definitive data exist about the management of severe peritonitis with the open abdomen. Robledo et al. compared open versus closed abdomen procedures in 40 patients with severe secondary peritonitis; no significant differences in mortality rates were found (55% open vs. 30% closed). The study was interrupted at the first interim analysis for high relative risk and odds ratios for death in the open group (1.83 and 2.85, respectively) [16]. However, the TAC technique that was selected as the “intervention” would be relatively contraindicated in current OA management. Some other cohort studies showed the effectiveness of OA technique in treating severe peritonitis. At present, however, no definitive data from randomized trials exist.

### Open abdomen in vascular emergencies

*The open abdomen should be strongly considered following management of hemorrhagic vascular catastrophes such as ruptured abdominal aortic aneurysm (grade 1C).*

*The open abdomen should be considered following surgical management of acute mesenteric ischemic insults (grade 2C).*

The ACS has been well described in the setting of ruptured abdominal aortic aneurysm (rAAA) [17]. Rupture of aortic as well as iliac or visceral aneurysm often results in life-threatening hemorrhagic shock. The combination of severe shock and massive resuscitation contributes to retroperitoneal, mesenteric, and bowel wall edema and production of ascites that can increase abdominal pressure and lead to ACS. Intra-abdominal hypertension occurs in up to 50% of patients following AAA repair, and ACS occurs in 8–20%. Mortality after rAAA is as high as 30–50%; of note, mortality is generally twice as high among patients who develop ACS compared with those who do not [18].

Consequently, prevention of ACS, if possible, would be of tremendous benefit to the patient.

In prospective non-randomized studies, the incidence of ACS is reduced when prophylactic OA is employed [19]. Unfortunately, selection criteria for employing OA are not well defined; the surgeon might consider inability to close the fascia without tension; use of aortic balloon occlusion catheter; and preoperative blood loss >5 L [19, 20]. Such criteria should prompt the surgeon to consider temporary OA utilization. When the abdomen is closed primarily, postoperative monitoring of IAP is recommended, with vigilance for ACS as reflected by elevated airway pressures, reduced cardiac output, or oliguria. Concerns for infection of aortic grafts with OA are allayed by existing data, indicating a relatively low rate [21]. Patients are often selected for endovascular repair (EVAR) of rAAA if they have less hemodynamic compromise. Although it is less common, ACS still occurs after EVAR [17]. The major risk factor appears to be massive resuscitation. These patients should have vigilant monitoring for elevated IAP and the onset of ACS.

Mesenteric ischemia may result from arterial (thrombotic, embolic, or low perfusion) or venous (venous thrombosis) insults. Fundamental principles of management include making the diagnosis, restoration of intestinal perfusion, and assessment of bowel viability with resection as necessary. The bowel ischemia leads to bowel wall and mesenteric edema, as well as ascites production; reperfusion of the bowel can exacerbate bowel edema and ascites and thus increase risk of ACS. For this reason, OA use should be considered following restoration of perfusion in a patient with acute mesenteric ischemia. As there are no reliable independent predictors of ACS in this setting, the surgeon should assess bowel swelling and the patient’s physiology to make this decision [22, 23]. Another reason to consider temporary OA following mesenteric ischemia is to facilitate second-look laparotomy to assess bowel viability and perform bowel anastomosis as needed [24]. Bowel resection is much less common in the setting of venous thrombosis than arterial occlusion, so the patients with mesenteric venous thrombosis probably do not require OA as often as those with acute arterial occlusion [25]; although, IAP should be followed.

### Open abdomen in pancreatitis

*In patients with severe acute pancreatitis unresponsive to step-up conservative management, surgical decompression and leaving the abdomen open is effective in treating abdominal compartment syndrome (grade 2C).*

*Leaving the abdomen open after surgical necrosectomy for infected pancreatic necrosis is not recommended except in those situations at high risk of abdominal compartment syndrome (grade 1C).*

Acute pancreatitis (AP) is a mild self-limiting disease in the majority of cases, even though the 15% of patients with AP progress to severe disease identified by development of persistent organ failure [26]. Multiple organ failure (MOF) is the factor mainly associated to mortality in AP, as a counterpart in absence of organ dysfunction or if it transient the risk of dying is very low [27–29]. However, in those with severe AP, MOF develops generally early, with over half of the patients exhibiting organ dysfunction’s signs at hospital admission and in any case, most part of them develops within the first 4 days after admission [28, 30]. More than half of the deaths happen within the first week from onset of AP and generally within a week after MOF first symptoms [31]. Principal treatments of MOF are support therapies: vasopressors, fluid replacement, and renal replacement therapy and mechanical ventilation if indicated. During AP, IAH/ACS may aggravate MOF, and therefore, constant IAP measurements are crucial to identify patients with high risk of developing ACS [32]. ACS should be prevented and treated, whenever possible, with non-operative management. Surgical decompression is the last but the most effective tool to decrease the IAP, and it should not be postponed if the patient presents ACS manifestation [5, 33].

In the event of AP, the risk to develop subsequent infections (i.e., bacteremia, pneumonia and infection of pancreatic or peripancreatic necrosis) is increased. The first week of illness is crucial for the extra-pancreatic infection occurrence, whereas pancreatic necrosis usually becomes infected later [34]. Some factors are associated to an increased risk of infected necrosis: the presence of organ failure, early bacteremia, and the extent of pancreatic necrosis [34]. Surgical necrosectomy is the last resort if more conservative management including percutaneous drainage failure [35]. Patients with persistent organ failure complicated with infected pancreatic necrosis face a very high mortality risk [36].

### Optimal technique for temporary abdominal closure

*Negative pressure wound therapy with continuous fascial traction is suggested as the preferred technique for temporary abdominal closure (grade 1B).*

*Temporary abdominal closure without negative pressure wound therapy (*e.g.*, mesh alone, Bogota bag) whenever possible should NOT be applied for the purpose of temporary abdominal closure, because of low delayed fascial closure rate and being accompanied by a significant intestinal fistula rate (grade 1B).*

*There is inadequate evidence to make a recommendation regarding use of negative pressure wound therapy in combination with fluid instillation in patients with temporary abdominal closure (NOT GRADED).*

The perceived indications and subsequent treatment choices in managing OA differ among surgeons. The existing techniques result in different risk of entero-atmospheric fistula (EAF) and the different rate of delayed fascial closure. Overall, 74 relevant studies exist for a total of 4358 patients: 3461 (79%) with peritonitis. The described OA indications are considerably different. Thirty-eight out of 78 series described negative pressure wound therapy (NPWT) TAC systems. NPWT with a dynamic component (mesh-mediated fascial traction or dynamic sutures) gives the best results in terms of delayed fascial closure, but dynamic sutures result more often in fistula. NPWT without a dynamic component (Barker’s VAC or commercial products) for the use of temporary fascial closure has a moderate delayed fascial closure rate and a fistula rate similar to mesh closure without NPWT.

Several TAC techniques exist that could be used alone or combined together. Six-eight series reported about one TAC technique. Ten series described patients managed with combined TAC systems. NPWT was used alone in 32 studies [37–68], and in 6 studies, NWPT is combined with fascial traction (mesh or sutures) [69–74] and eight series described the use of meshes (non-absorbable and/or absorbable) [75–81]. Six series reported about the Bogota-bag use [75, 82–86]; five, about Zipper [87–91]; and other five, about dynamic retention sutures [92–96]. Two more series described loose packing [97, 98]. Lastly, the Wittmann patch was used in one series [99]. The remnant three series applied different TAC systems [82, 100, 101]. The delayed fascial closure rate ranged from 3.2 to 100%.

Twenty-two series were prospective, and ten out of them described NPWT (608 patients) showing a weighted fascial closure rate of 53.9% and an EAF rate of 9.8%. The four prospective series on NPWT with fascial traction (411 patients) showed a weighted fascial closure rate of 77.8% and an EAF rate of 4.3%. Including retrospective studies data per closure type are in line with the aforementioned results. With the highest weighted fascial closure rate for NPWT with fascial traction (73.1%) and dynamic retention sutures (73.6%). TAC using a mesh or zipper showed the lowest delayed closure rates (34.2 and 34.0% respectively). Nine series were not exhaustive in describing eventual fascial closure attempts [16, 45, 75, 81, 87, 89, 98, 102, 103].

#### Is there a role for NPWT with fluid instillation?

There are no series published on the use of NPWT with instillation in situations of TAC in non-trauma patients or in trauma patients. Recently, a systematic review performed by an expert consensus group has been published underlining the need of more evidence to support the fluid instillation and giving no recommendation of its use in abdominal wound [104].

### Planning re-exploration before definitive closure

*In critically ill non-trauma patients with open abdomen, once any requirements for on-going resuscitation have ameliorated, early re-operation with the intention of closing the abdomen should be given a high priority (grade 1C).*

*In critically ill patients with open abdomen, re-laparotomy with concern for ongoing ischemia/contamination re-operation should be conducted no later than 24–48 h after the index operation, with the duration from the index operation shortening with increasing degrees of patient non-improvement and hemodynamic instability (grade 1C).*

A related question for clinicians is when to re-operate (if ever) for the sole purpose of “revise” when there is recognition that closing an abdomen will not be possible. This question may be further conceptually complicated in an attempt to distinguish indications to re-operate because the patient is not improving or deteriorating and there is fear that contamination or ischemia is ongoing and those cases of non-improvement or only modest improvement in whom there is operation intention to “wash” the peritoneal cavity and to “change” the TAC dressing or device. No RCTs or meta-analyses examining the timing of re-operation in OA patients exist. Guidelines and review papers did not generally discuss timing of re-operation [8, 105]. In the position paper of the WSES, it is recommended that as a general principle, patients should be taken back to the operating room at 24–48 h after the initial surgery [2]. Other expert opinions come from the survey of Trauma Association of Canada in 2006, and the majority of responders indicated the best timing included between 24 and 72 h [106, 107]. Pommerening et al. utilized the American Association for the Surgery of Trauma (AAST) Open Abdomen Registry to evaluate time to the first re-operation on trauma OA patients as a predictor of primary fascial closure using a hierarchical multivariate logistic regression analysis [108]. Adjusting for other factors, including resuscitation volumes, increasing delay to the first re-operation was associated with a decreased likelihood of primary fascial closure (PFC), with a 1.1% decrease in PFC rates for every hour after 24 h from the index operation [108]. Further, there was a trend (95% CI 1.0–3.25 OR) of increased complications in patients having the first re-operation after 48 h [108].

It should be clearly understood however that extrapolation of these findings regarding the timing of re-operation in trauma patients might not be directly applicable to non-trauma patients with OA. It is becoming apparent that infected and non-infected patients with auto-activation of the immune responses leading to multi-organ dysfunction syndrome (MODS) and MOF have more fundamental differences than previously appreciated [109]. Fundamental evidences from basic science are emerging justifying the OA in critically ill/injured patents in order to manipulate the systemic immune response and ameliorate the bio mediator burdens of catastrophic illness [110–113]. There are also newly described populations of fully mature indwelling peritoneal macrophages that migrate locally within the peritoneal cavity within an hour of injury [114]. Whether mechanically removing such cell populations through scheduled “wash-outs” is beneficial or harmful is a completely unstudied question. Thus, the timing of re-operation is more complex in non-trauma patients and urgently requires further study. Lastly, in critically ill patients with an OA, re-laparotomy with the intention of cleaning or “washing-out” the abdomen has an unknown priority and should be subjected to future randomized study.

### Best timing to definitively close an open abdomen

*Fascia should be closed as soon as possible (grade 1C).*

*Acidosis (pH <7.25), hypothermia (temperature <34 °C), and coagulopathy (TEG, INR) are not predictive of the need for maintaining the open abdomen in non-trauma patients (grade 2A).*

*The abdomen should be maintained open in non-trauma patients if the source of contamination persists, if a condition of hemodynamic instability persists meaning in the presence of an on-going fluid resuscitation or vasopressor support necessity, if a deferred intestinal anastomosis is needed, if there is the necessity for a planned second look for ischemic intestine, and lastly if there are concerns about abdominal compartment syndrome development (grade 2C).*

*Early fascia closure (within 7 days) should be the strategy for management of the open abdomen once the source control has been reached, the severe sepsis has been controlled meaning that the patient is hemodynamically stable and the hypoperfusion has been definitively corrected, no further surgical re-exploration is needed, and there are no concerns for abdominal compartment syndrome (grade 2C).*

The early definitive abdominal closure is the first goal to achieve in order to reduce the OA complications rate [115], (i.e., EAF, fascial retraction with loss of abdominal wall domain, and incisional hernias) [115, 116]. The primary closure rates have a bimodal distribution, with early closure depending on postoperative intensive care management and delayed closure depending on the choice of the TAC technique [117]. Mortality, complications, and length of stay were compared between early and delayed fascial closure in a meta-analysis [118]. 3125 patients were included and 1942 (62%) successfully achieved early fascial closure. Early fascial closure is a factor significantly associated with a reduced mortality (12.3 versus 24.8%, RR 0.53, *P* < 0.0001) and complication rate (RR, 0.68, *P* < 0.0001). Early fascial closure is commonly performed within 4–7 days of the initial laparostomy [13]. No major technical difficulties are described to obtain primary fascial closure within few days from the index operation. Patients having abdominal sepsis are less likely to achieve an early fascial closure [119] and therefore should have closure attempts performed as soon as possible after severe abdominal sepsis is controlled [120].

### Best solution to definitively close an open abdomen

Often the OA, particularly if prolonged, results in fascia retraction and consequently in large abdominal wall defects that require complex abdominal wall reconstruction. Moreover, the situation is often complicated by a contaminated field [121] with high risk of infections and wound complications, such as wound infections, seromas, fistula formation, recurrence of the defect, and mortality [122–124].

#### Non-mesh-mediated techniques

*Primary fascia closure is the ideal solution to restore the abdominal closure (grade 2A).*

*Component separation is an effective technique; however, its early use is NOT recommended in fascial temporary closure. It should be considered only for definitive closure or reconstructive interventions (grade 2C).*

*Planned ventral hernia (skin graft or skin closure only) remains an option for complicated open abdomen (*i.e., *in the presence of entero-atmospheric fistula or in cases with a protracted open abdomen due to underlying diseases) or in those low-resource setting where no other facilities are present (grade 2C).*

Abdominal component separation is most commonly considered an elective procedure for ventral hernia repair [118]. One important technique described for the reconstruction of the abdominal wall is the component separation. The technique of anterior component separation consists in a relaxing incision made in the aponeurosis of the external oblique muscle, a separation of the external and internal oblique muscle and the incision of the rectus fascia to achieve the advancement of the abdominal wall to cover the defect. This technique has been well studied and described in elective giant ventral hernia repair, and it provides an effective technique with a recurrence rate of 16% [125, 126] but a very relevant complication rate of 50%. Other surgical techniques that have been described include the posterior component separation: the rectus sheath is opened and the posterior rectus fascia and rectus muscle are separated. At the lateral margin of the rectus muscle, the aponeurosis of the transverse abdominis muscle is incised with the separation of the internal oblique muscle from the transverse abdominis muscle.

However, the use of abdominal component separation technique was recently described in acute fascia closure after open abdomen in a small case series by Rasilainen et al. [127] with 75% of primary fascia closure. At present, there is not enough evidence to support component separation in the acute setting due to the related high morbidity and the fact that these techniques can only be performed on a patient once, so that if ill timed, future options are not available. Therefore, a valuable alternative option for closure of the open abdomen remains the planned ventral hernia: its main goal is to cover abdominal viscera to prevent complications such as EAF. The abdominal wall defect could be closed only with skin suture and or a skin graft put on the underlying granulating tissue creating a planned laxity. After physiologic recovery and a significant period of scar and adhesion maturation, the complete restoration of the patient’s abdominal wall through reconstructive techniques can be undertaken as an elective procedure.

#### Mesh-mediated techniques

*A fascial bridge using prosthetic mesh (polypropylene, polytetrafluoruroethylene (PTFE) and polyester products) should not be recommended to achieve definitive fascial closure in patients with open abdomen and should be placed only in patients without other alternatives (grade 1B).*

*Biologic meshes are reliable for definitive abdominal wall reconstruction in the presence of a large wall defect, bacterial contamination, comorbidities, and difficult wound healing. NPWT can be used combined with biologic mesh to facilitate granulation and skin closure (grade 2B).*

*Non-cross-linked biologic meshes seem to be preferred in sublay position when the linea alba can be reconstructed. Non-cross-linked biologic mesh is easily integrated, with reduced fibrotic reaction and lesser infection and removal rate (grade 2B).*

*The long-term outcome of a bridging non-cross-linked biologic mesh is laxity of the abdominal wall and a high rate of recurrent ventral hernia. In the bridge position (no linea alba closure), cross-linked biologic meshes maybe associated with less ventral hernia recurrence (grade 2B).*

Two meta-analyses exist on BP in abdominal wall defect. The first, by Sharrock et al. investigated the management and closure of OA in trauma patients [128]. Among the included studies, the point estimate recurrence rate of ventral hernia after 1 year of BP positioning was 51%. However, the authors highlighted the small number of included studies and their poor quality; moreover, as above mentioned, great differences exist between trauma and septic patients and great caution should be addressed in interpretation of this result. A systematic review and meta-analysis by Atema et al. [129] investigated the utilization of BP in abdominal wall reconstruction. They clearly stated that the poor quantity and quality of available data strongly limits taking a clear message from the results. Biological material in infected fields had a recurrence rate of 30% compared with 7% of synthetic material, but data were derived from a single study and does not justify the use of synthetic materials, especially as a bridge position after OA.

The “bridging” technique refers to using some mesh (either prosthetic or biologic) to physically interpose between native abdominal wall fascia that either cannot or should not be primarily opposed. Thus, such fascial defects can be closed with a mesh in a bridging position. In general, non-absorbable synthetic materials (i.e., polypropylene mesh) reinforce any fascial repair through a combination of mechanical tension and intense inflammatory reaction, resulting in the entrapment of the mesh into scar tissue. However, in a bridging position, there is no native tissue to protect viscera from the mesh and thus, the persistent inflammatory response combined with the contaminated field may induce local side effects such as adhesions, erosions, and fistula formation [130–135]. International guidelines on emergency repair of abdominal wall hernia therefore do *not* recommend the use of synthetic meshes in contaminated fields [136].

Biological prosthesis (BP) has been designed to perform as permanent surgical prosthesis in the abdominal wall repair, minimizing mesh-related complications [137]. The rationale of their usage in OA is based on the premise that the implantation of a biologic material triggers a cascade of events leading to new healthy tissue deposition and prosthesis remodeling. The presence of vital tissue therefore allows for perfusion and a native immune response preventing mesh infection and abscess formations. The ideal BP will also maintain mechanical characteristics of a synthetic mesh with a sufficient mechanical strength to withstand the physiological and anatomic stresses of the human abdominal wall. Such an ideal BP should also tolerate adjunctive NPWT to facilitate wound healing, granulation, and skin closure [100, 138].

Discordant data have been published about the use of BP to bridge a wide defect of the abdominal wall. The evidence is limited with few studies, all non-randomized, and with an overall small number of cases. Further among heterogeneous patients reported, recurrence rates have ranged between 0 and 100% [139–152]. When used as a bridge to close the fascia defect, the reported recurrence rate in a large retrospective series was >80% [153]. Another study by Booth and colleagues compared primary fascia closure with mesh reinforcement with the use of the mesh as a bridge and demonstrated a higher recurrence rate in the mesh in a bridge position (8 vs. 56%, *p* < 0.001) [154].

Several studies investigated the best anatomical position in terms of BP function, but were not specifically focused on OA reconstruction. Nonetheless, evidence, including that from randomized trials, suggest that implanting the BP in the sublay position results in a lower recurrence and complication rate [155–157]. However, it should be stressed that the data included was not specific for the OA situation and the heterogeneity among patients and indications was very high, resulting in a poor level of evidence.

Two meta-analyses exist on BP in abdominal wall defect. The first, by Sharrock et al. investigated the management and closure of OA in trauma patients [128]. Among the included studies, the point estimate recurrence rate of ventral hernia after 1 year of BP positioning was 51%. However, the authors highlighted the small number of included studies and their poor quality; moreover, as above mentioned, great differences exists between trauma and septic patients and great caution should be addressed in interpretation of this result.

A systematic review and meta-analysis by Atema et al. [129] investigated the utilization of BP in abdominal wall reconstruction; the poor quantity and quality of available data strongly limits the results. Biological material in infected fields had a recurrence rate of 30% compared with 7% of synthetic material, but data were derived from a single study and does not justify the use of synthetic materials, especially as a bridge position after OA.

In conclusion, no definitive evidence-based conclusions could be obtained currently from the literature. The available evidence is really weak: most of the cited meta-analysis included rather poor quality retrospective case series. There is also great heterogeneity among the indications for mesh implantation, the anatomic positioning of the mesh, and the type of mesh. This further weakens the quality of the evidences. Thus, well-performed randomized trials comparing different type of meshes and the techniques of mesh positioning are urgently required.

### Best treatment for open abdomen and entero-atmospheric fistulas

*Several clinical circumstances may contribute to the development of entero-atmospheric fistula and few risk factors may predict its development. Awareness of this complication and avoidance of contributing conditions for its development are mandatory; moreover, preemptive measures are imperative (grade 1C).*

*The management of entero-atmospheric fistula should be personalized according to standard classification and grading system. Current different classification schemes echo the problematic and challenging issues related to their management (grade 1C).*

*The caloric intake and protein demands of patients with entero-atmospheric fistula increase; the nitrogen balance should be corrected and protein supplemented. Nutrition should be started immediately upon recognition of entero-atmospheric fistula (grade 1C).*

*Entero-atmospheric fistula effluent isolation is essential for proper wound healing. Separating the wound into different compartments in order to facilitate the collection of fistula output is of paramount importance (grade 2A).*

*Many methods for wound care exist; however, in the presence of entero-atmospheric fistula in an open abdomen, negative pressure wound therapy makes effluent isolation feasible and wound healing conceivable (grade 2A).*

*Definitive management of entero-atmospheric fistula should be delayed to after the patient has recovered and the wound completely healed (grade 1C).*

Enteric fistula is a severe complication following abdominal surgery. The opening of a fistula onto dehisced wound therefore exposing and communicating the bowel and its effluent to the atmosphere is defined as EAF. The incidence of EAF varies from 4.5 to 25% in the trauma setting [158] and from 5.7 and 17.2% in non-trauma patients [105]. The presence of this complication dramatically increases considerably mortality, length of stays, and costs [159].

Many factors may contribute to the development of EAF. All linked as a “vicious cycle”: the lack of overlying soft tissue, with its blood supply, precludes spontaneous healing and the exposed viscera predispose to additional disruptions in the gastrointestinal tract. EAFs may result from various etiologies: anastomotic dehiscence or disruption, iatrogenic injury during dissection or inappropriate handling, and presence of synthetic prosthetic material (i.e., mesh) and from the prolonged exposure of bowel [160–163]. ACS and severe IAH may result in reduced bowel blood supply and therefore contribute to EAF development [68]. A prospective analysis of 517 trauma emergency laparotomies showed that large bowel resections, large volume fluid resuscitation (>5 L/24 h), and increased number of re-explorations were significantly associated with an increased incidence of EAF [158]. Preemptive measures could be undertaken in order to prevent this complication: early abdominal wall closure, bowel coverage with omentum or skin, and no direct application of NPWT on the viscera are some of these measures [112, 164, 165].

**Fig. 1:**
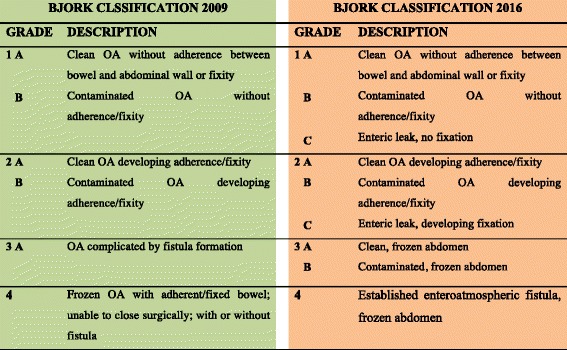
Open Abdomen classification according to Bjork et al. [168]

Several classifications and grading systems of EAF exist. Schein and Decker proposed in 1991 a grading system based on the fistula location. Grade IV indicates a fistula related to large abdominal wall defects with grades IVa and IVb indicating the site of the fistula in regards to its location [166]. EAF can be classified based on the fistula effluent output: low (<200 ml/day), moderate (200-500 ml/day), and high (>500 ml/day) [167]. Bjork et al. proposed a classification based on the presence of adhesions of the bowel in the setup of the open abdomen as well as the association to the fistula formation (Fig. 1), and this was later adapted by WSACS [168]. Di Saverio et al. proposed a comprehensive classification based on the combination of different criteria as anatomical location, output, exposure, and number of fistulas [169]. As a general principle, a single, superficial fistula located in the lower GI tract with a low output has a higher probability of spontaneous closure rather than multiple fistulas deep in the wound with high output [169, 170]. According to this principle, the management should be tailored to each clinical situation and individualized accordingly. In conclusion, the presence of several different classifications represents the true difficulties in the management of EAF in OA. Level of evidence is poor and many recommendations are based on expert opinion suggestions.

EAF is a poorly predictable and, above all, avoidable complication. When patients develop EAF, an accurate and tailored management scheme should be adopted. Nutrition plays a key role in the management of these patients and should be always kept in mind as a fundamental part of the treatment. The open abdomen strategy may result in fluid and electrolytes loss resulting in acid-base derangements [8]. The anatomy and the characteristics of the EAF(s) should be defined in order to plan the best treatment option [171]. Parenteral nutrition (TPN) should be started immediately after the patient resuscitation. Enteral nutrition in OA patients has been well studied demonstrating a reduction in infectious complications preserving the intestinal mucosal barrier and its immunological function [172–174]. Enteral nutrition in patients with an EAF is has but may increase fistula output. Only small series of patients with EAF treated with EN exists; therefore, no strong evidence can support these treatments and further studies are needed [175, 176]. The use of octreotide analogs is controversial. No evidence exists about the use of somatostatin and octreotide in managing of EAF. Few studies suggest that octreotide may reduce fistula output by diminishing GI secretions [177] while others argue their benefit due to this agents’ reduction in splanchnic blood flow and reduction in immune function [178, 179].

The main goal in the management of EAF should be the closure of the fistula. Differently from common GI fistulas, the EAF is not a true fistula since a fistula tract does not exist. The lack of surrounding tissues prevents the spontaneous closure. The goal of the treatment should be focused on trying to isolate the fistula effluent and enhancing the formation of granulation tissues surrounding it. Several different techniques were described and proposed in the literature to control and treat EAF, and some attempts to standardize its management exist [169, 170]. A patient diagnosed with EAF in the setup of OA should be treated by medical personnel familiar with this complication and its consequences.

Accurate fistula definition and anatomy should be made. Sepsis control and management is important. Diversion of the fistula output in order to maintain clean the peritoneal cavity is mandatory. Fistula effluent should be measured in order to facilitate fluid balance and to ensure skin protection from its digestive nature on the skin. This will enhance and allow better patient care and mobility.

Several different dressing and techniques were described for the management of EAF, each one with relatively small case series and discordant results with a consequent poor level of evidence [162, 170, 180–183]. Proposed treatments vary from primary suture and fibrin glue for small exposed distal fistula to a fistula suspension fixating the fistula edges to the skin. Several variants of NPWT with devices for fistula isolation and diversion were described with promising outcomes.

The several techniques are described in detail elsewhere and are not in the scope of the current position paper [170]. The described method to manage NPWT in patients with EAF in the setup of OA should be applied depending on surgeon preference, skills, and expertise and according to hospital facilities and material availability. Generally, negative pressure wound therapy, with specifically described variants, is the most accepted technique. EAF isolation and proper wound management will enable skin grafting and converting EAF to a more controllable one with ease of applying effluent collection bag. The definitive treatment, i.e., closure of the fistula and repairing the abdominal wall defect should be postponed at least 6 months and only after the patient and the wound healed completely.

### Nutritional support

*Open abdomen patients are in a hyper-metabolic condition; an immediate and adequate nutritional support is mandatory (grade 1C).*

*Open abdomen techniques result in a significant nitrogen loss that must be replaced with a balanced nutrition regimen (grade 1C).*

*Early enteral nutrition should be started as soon as possible if the gastrointestinal tract allows (grade 1C).*

*Enteral nutrition should be delayed in patients with high output fistula with no possibility to obtain feeding access distal to the fistula (grade 2C).*

*Oral feeding is not contraindicated; whenever its possible, it could be started as soon as the patient is able to eat (grade 2C).*

The hyper-catabolic state of critically ill patients is associated with muscle proteolysis, acute protein malnutrition, immune function impairment, and subclinical development of MOF. Several studies clearly demonstrated malnutrition as a fundamental risk factor associated to poor outcomes during hospital stay [184]. Furthermore, in a critically ill patient, OA leads to significant nitrogen loss estimated to be 2 g per liter of abdominal fluid output. This issue requires adequate consideration and an adjusted integration [185]. For this reason, the measurement of the abdominal fluid loss is mandatory [185]. This loss in nitrogen and protein is ulterior greatly increased in the presence of EAF. A particular attention must be given to this critical aspect because patients with OA are the sickest, most inflamed, and subsequently most hyper-metabolic among surgical patients. During the OA patient management, once the resuscitation is almost completed and the GI tract allows it, EN should be started as soon as possible. Thus, it will bring beneficial effects for the patient as faster fascia closure and lower pneumonia and fistula rate [173, 186, 187]. If malnutrition occurs, mucosal atrophy and malabsorption are among the earliest consequences. Gut-associated lymphoid tissue seems to be diminished, and as a consequence, it can increase the risk for disseminated infection due to bacterial translocation through the intestinal wall [188]. EN helps in maintaining gut mucosal barrier in good shape and function; as a consequence, it has been demonstrated to enhance immunity and IgA secretion, to prevent muscle atrophy, and lastly to decreases systemic inflammation and oxidative injury [188, 189]. Early EN within the first 24–48 h is demonstrated to improve wound healing, decrease catabolism, preserve GI tract integrity, and finally, it reduces complications, length of hospital stay, and costs. Compared to TPN early EN decreases septic complications especially in abdominal trauma and traumatic brain injuries. A retrospective, single-institution study comparing DCS interventions with open abdomen performed to treat ACS, 43 patients underwent early (<4 days) and 35 late (>4 days) EN. Early EN significantly increased primary closure (74% vs. 49%), reduced the fistula rate (9% vs. 26%) with no difference in infections and but with a significant reductions in hospitalization costs [186].

### Patient mobilization

*To date, no recommendations can be made about early mobilization of patients with open abdomen.*

Patients with an open abdomen generally should not be mobilized out of bed until their abdomens are definitively closed, for risk of evisceration [190]. This statement was extrapolated from trauma literature [191]. However, prolonged bed rest is associated with significant increase in complication rate. More recent attention has been focused on intensive care unit (ICU)-acquired weakness and the long-term adverse functional sequelae for ICU survivors, particularly in the physical domain and this has led to an increased interest in early mobilization in the ICU as a potential means of prevention [192–196]. The optimal timing for initiation of mobilization of patients with OA has yet to be defined. Early mobilization is currently defined as occurring within the first 2 to 5 days of ICU admission [197].

Patients with open abdomen managed with NPWT however, may be mobilized by active or passive transfer. Further research must occur to provide the rationale to early mobilization prior to definitive abdominal closure.

## Conclusions

Management of the open abdomen remains a very controversial domain, in which many techniques are still debated. Many important issues remain to be addressed through carefully designed and rigorously conducted studies. Until better data is available, the use of the OA should be carefully tailored to each single patient taking care to not overuse this effective tool. Every effort should be exerted to attempt abdominal closure as soon as the patient can physiologically tolerate it. Finally, all the precautions should be considered to minimize the complication rate.

## Abbreviations

AAST: American Association for the Surgery of Trauma
ACS: Abdominal compartment syndrome
AP: Acute pancreatitis
BP: Biological prosthesis
EAF: Entero-atmospheric fistula
EN: Enteral nutrition
EVAR: Endovascular repair
GRADE: Grading of Recommendations Assessment, Development and Evaluation
IAH: Intra-abdominal hypertension
IAP: Intra-abdominal pressure
INR: International Normalized Ratio
MODS: Multi-organ dysfunction syndrome
MOF: Multiple organ failure
NPWT: Negative pressure wound therapy
OA: Open abdomen procedure
PTFE: Polytetrafluoruroethylene
rAAA: Ruptured abdominal aortic aneurysm
RCT: Randomized controlled trial
TAC: Temporal abdominal closure
TEG: Thromboelastography
TPN: Parenteral nutrition
WSACS: The Abdominal Compartment Syndrome
WSES: World Society of Emergency Surgery
